# Supplementary material for: Becoming Safe, Legal, Mature, Moderate, and Self-Reflexive: Trajectories of Drinking and Abstinence among Young People
Source: Int J Environ Res Public Health. 2022 Mar 17;19(6):3591. doi: 10.3390/ijerph19063591 (PMC8953176; doi:10.3390/ijerph19063591)
Supplement: Supplementary file 1 [file ijerph-19-03591-s001.zip › ijerph-1624252-supplementary.pdf]

International Journal of
Environmental Research and Public Health

Supplementary Material 1. Table of participants

Table S1. Descriptives of participants

	N	%
Gender		
Women	19	68
Men	9	32
Age groups at Wave 1		
15–17	12	43
18–19	16	57
Background		
Born in Sweden	19	68
Parents born abroad	7	25
Born abroad	2	7
Social class[a]		
Lower	9	32
Middle	11	39
Higher	8	29
Area[b]		
Large city	2	7
Medium-sized town	13	46,5
Small town	13	46,5
Socioeconomic status of area[c]		
Low status	11	39
Middle	9	32
High status	8	29
Total	28	100

[a] Based on categorisation of parents' occupations.

[b] Based on number of inhabitants in the municipality of residence. Large city ≥ 500,000, Middle town 50,000–499,999, Small town < 50,000.

[c] Based on categorisation of share of adult population with longstanding social welfare, average income of population, and ill-health rate of the municipality of residence.

Supplementary Material 2. Thematic semi-structured interview guide: Why are young people drinking less than earlier?

Theme 1. Information about the study and informed consent to participate

Theme 2. Background information: age, living situation, studies/work, family situation, pastime activites

Theme 3. Relationship to alcohol

    If drinker: Description of settings, situations, frequency, amount, beverages of preference, activities, company

    If non-drinker: Description of pastime activities, settings, situations, company

Theme 4. Friends´, parents' and siblings' alcohol practices, attitudes and supervision

Theme 5. A typical occasion of having fun (with or without alcohol)

Theme 6: Alcohol in relation to a) friendship, b) romantic relationships, c) health and well-being

Theme 7. Alcohol and gender norms

Theme 8. Alcohol and/or a) sports and exercise, b) use of social media, c) gaming, d) gambling, e) tobacco, f) drug use

Theme 9. Motives for drinking or non-drinking

In wave 2 and 3, follow-up questions were asked concerning changes in life situation (studies, work, change of residence etc.) and approaches to alcohol, based on the same themes and previous interview answers

Supplementary Material 3. Figure S1 of trajectories, actancts and chains of translations

Trajectory	Social, emotional and material actants	Chain of translation
Drinking becomes translated from unsafe to safe assemblages	**Main actant: Drinking place**<br>Unsafe assemblage: private home parties, underaged drinking, lack of control, vulnerability, illegal alcohol; *alcohol central*<br>Safe assemblage: public drinking venues, legal age drinking, control, absence of risk, regulated alcohol; *alcohol peripheral*	Increasing alcohol consumption
Drinking becomes translated from illegal to legal assemblages	**Main actant: Age limit**<br>Illegal drinking assemblage: underage, minor, no rights, immaturity, abstinence<br>Legal drinking assemblage: adult, rights, maturity,access, trust from parents, moderate drinking as a natural step towards becoming an adult	Increasing alcohol consumption
High-performing assemblages exclude drinking or becomes translated to include drinking for pleasure	**Main actant: Performance**<br>High-performing assemblage: self-control, responsibility, expectations, success, high grades, elite sports, abstinence<br>Assemblage of pleasure: having fun, well-being, enjoyment, socialising, moderate drinking	Maintained abstinence or increasing alcohol consumption (from abstinence to moderate)
Heavy drinking becomes translated from immature to mature assemblages	**Main actant: Intoxication**<br>Immature assemblage: partying as "mandatory", drinking heavily, unhealthily and irresponsbily part of being young<br>Mature assemblage: controlled and moderate drinking, diverse options, staying home is acceptable, health concerns, awareness of risks, cautiousness	Decreasing alcohol consumption
Abstention is translated from authoritative assemblages to self-reflexive assemblages	**Main actant: Abstinence**<br>Authoritative assemblage: religious beliefs, parental rules, subcultural norms, temperance or political values<br>Self-reflexive assemblage: personal reasons based on authentic individual experiences and choices	Maintained abstinence
